# Improved Cryptanalysis and Enhancements of an Image Encryption Scheme Using Combined 1D Chaotic Maps

**DOI:** 10.3390/e20110843

**Published:** 2018-11-03

**Authors:** Congxu Zhu, Guojun Wang, Kehui Sun

**Affiliations:** 1 School of Information Science and Engineering, Central South University, Changsha 410083, China; 2 School of Computer Science and Technology, Guangzhou University, Guangzhou 510006, China; 3 School of Computer and Science, Liaocheng University, Liaocheng 252059, China; 4 School of Physics and Electronics, Central South University, Changsha 410083, China

**Keywords:** image encryption, chaotic cryptography, cryptanalysis, chosen-plaintext attack, image information entropy

## Abstract

This paper presents an improved cryptanalysis of a chaos-based image encryption scheme, which integrated permutation, diffusion, and linear transformation process. It was found that the equivalent key streams and all the unknown parameters of the cryptosystem can be recovered by our chosen-plaintext attack algorithm. Both a theoretical analysis and an experimental validation are given in detail. Based on the analysis of the defects in the original cryptosystem, an improved color image encryption scheme was further developed. By using an image content–related approach in generating diffusion arrays and the process of interweaving diffusion and confusion, the security of the cryptosystem was enhanced. The experimental results and security analysis demonstrate the security superiority of the improved cryptosystem.

## 1. Introduction

The transmission of a digital image from the public network is becoming more and more frequent nowadays. Consequently, it is urgent to guarantee the security and privacy of image transmission, especially for military images and some sensitive content images. As an essential technical means, image encryption approaches are particularly important in image communications. However, traditional cryptography cannot quickly encrypt images with large amounts of data. As traditional cryptography relies on the complexity of computation, it is not easy to generate a large number of keys quickly. In this application background, chaotic encryption is a good complement to traditional cryptography, especially in image encryption. As chaotic signals have some excellent characteristics required by cryptography, chaotic systems have become a fine tool for information encryption [1], especially for image encryption applications. Due to this, chaotic systems have been widely used in designing image encryption algorithms. Entropy is an important measure of the chaotic characteristics of dynamical systems. Entropy, chaos and information theory are closely related [2,3,4]. 

Among many chaos-based algorithms for encrypting an image, the permutation and diffusion (PD) structure encryption algorithm, proposed by Fridrich [5], has become a typical model. This structure consists of a permutation (i.e., pixel position scrambling) procedure and a diffusion (i.e., pixel value alteration) procedure. Based on a typical model, researchers have tried many different ways of improving innovation. Some studies have proposed different image permutation strategies [6,7,8,9,10,11,12]. Some researchers have proposed novel image diffusion techniques [10,13,14,15,16,17]. Many researchers have attempted to improve the performance of image encryption systems through other improvements. References [18,19,20,21,22,23,24] improve the performance of secret key streams through a new chaotic system model. References [25,26,27,28,29] improve the anti-attack performance of a cryptographic algorithm by introducing a plaintext-related mechanism in generating the key streams. References [30,31,32,33,34,35,36] introduce the DNA coding principle in bioinformatics to enhance the security of the algorithms. References [37,38,39,40,41] focus on improving the speed of image encryption algorithms through comprehensive means. In References [42,43,44], the S-boxes are applied to the design of efficient image encryption algorithm and combined with transformation technology, the performance of the image encryption algorithm is improved. In References [45,46,47], wavelet analysis technology is introduced into the field of image encryption and the ideas are novel. In References [48,49], fractal analysis technology is investigated, which is related to chaos and has a good application potential in image encryption. 

Another research direction closely related to encryption is cryptanalysis. The goal of cryptanalysis is to find a way to decipher secret keys or plaintext without knowing the secret keys of encryption systems [50,51,52,53]. Cryptanalysis can also find out flaws in encryption algorithms and can help cryptographic system designers to improve the security performance of cryptographic algorithms, which can avoid losses caused by potential vulnerabilities and make valuable contributions to encryption. Hence, cryptanalysis can also promote cryptography. Recent cryptanalysis research shows that some chaos-based image encryption algorithms have some security flaws and the cryptosystems can be broken by using various attack methods. For example, we launched a chosen-plaintext attack [54] on the scheme in Reference [55]. Wu [53] broke the encryption scheme in Reference [56] by a chosen-plaintext attack. 

Except for the security performance, efficiency is another important issue of an image encryption scheme for practical applications. For this reason, image ciphers with a higher speed are consequently more desirable than those with a low speed. It is well known that low-dimensional discrete chaotic systems need less time than high-dimensional continuous-time chaotic systems to generate chaotic sequences of the same length. Therefore, using a low dimensional discrete chaotic system as a key generator of a cryptosystem has a higher speed. Furthermore, the complexity of discrete chaotic systems is much larger than those of the continuous-time chaotic systems [57,58,59] and the cipher image encrypted with a chaotic sequence of much larger complexity has a higher security. Therefore, using a 1D discrete chaotic system to encrypt color images, not only has the advantage of fast speed but also has the advantage of higher security. In Reference [60], Pak proposed a color image encryption scheme (denoted as Pak’s cryptosystem hereinafter) by using combined 1D chaotic maps. Pak’s system has the merits of a simple structure, high speed and a relatively high safety. It is a pity that Pak’s algorithm cannot resist the chosen-plaintext attack. To the best of our knowledge, so far, only Wang [61] and Chen [62] have done cryptanalysis on Pak’s scheme. Unfortunately, neither of the two previous analyses can crack all of the unknown parameters of Pak’s encryption scheme due to the difficulty of the comprehensive cryptanalysis. Therefore, the previous cryptanalysis is incomplete. Moreover, Wang’s cryptanalysis scheme has obvious problems and a very low efficiency, while Chen’s cryptanalysis scheme did not give the specific process of deciphering the permutation secret keys. In order to overcome the shortcomings of the above cryptanalysis work, this paper presents a more comprehensive and efficient cryptanalysis on Pak’s cryptosystem. With our improved cryptanalysis, both equivalent secret keys and all of the unknown parameters of Pak’s cryptosystem can be completely deciphered. 

Despite its security flaws, Pak’s encryption scheme still has many advantages to carry forward. Its design idea is clear and novel, and its efficiency is relatively high. Therefore, it is worth preserving these advantages and improving their defects. For this reason, this paper further proposes an improved enhanced color image encryption scheme, which includes both an image content–related approach in generating diffusion arrays and the process of interweaving diffusion and confusion.

The rest of this paper is organized as follows. Section 2 describes briefly Pak’s algorithm and the related cryptanalysis. The improved cryptanalysis and attacks on Pak’s algorithm are presented in Section 3. An enhanced encryption scheme is proposed in Section 4. Some experimental results and analysis for the enhanced scheme are given in Section 5. Finally, some concluding remarks are given in Section 6.

## 2. Description of Pak’s Scheme and the Related Cryptanalysis

**Figure 1:**
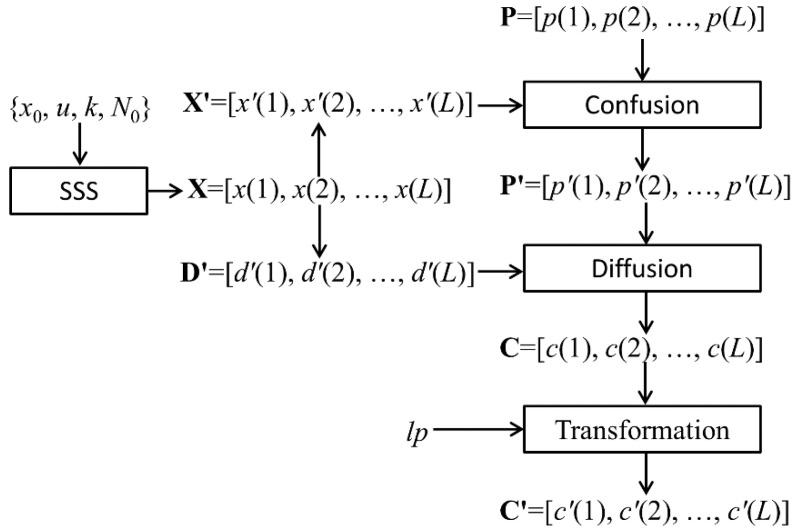
A flow chart of Pak’s encryption scheme.

Pak’s algorithm includes three processing stages. (1) Confusion: Pixel level permutation; (2) Diffusion: Pixel values encryption; (3) Linear transformation. Before the encrypting process, the 3D RGB color image with *M* row *N* columns is converted into a 1D pixel array **P** = [*p*(1), *p*(2), …, *p*(*L*)] according to the R, G, and B components successively, where *L* = *M* × *N* × 3. Each value of *p*(*i*) is an integer in the range [0, 255]. The flow of Pak’s encryption scheme can be visualized in Figure 1. Where, **P** is the plain image pixel array, and **C***′* is the final cipher image pixel array. SSS represents the combined Sine-Sine chaotic System. **X***′* is the permutation position array and **D***′* is the diffusion array. Both **X***′* and **D***′* are generated by chaotic sequences. 

### 2.1. The New Chaotic System 

The chaotic system adopted in Pak’s encryption scheme is a newly discovered chaotic map by using the chaotic sine map, which is expressed as

(1) $$x(n+1)=u\times \sin[\pi \times x(n)]\times 2^{k}-\left\lfloor u\times \sin[\pi \times x(n)]\times 2^{k}\right\rfloor \;$$

where, *u* is the control parameter of the system and {*x*(*n*), *n* = 0, 1, 2, …} is the output chaotic sequence with the initial value *x*(0) = *x*_0_. ⌊x⌋ is the largest integer that is smaller than or equal to *x*. System (1) is called a Sine-Sine system (SSS) [60], which is chaotic when *u* ∈ (0, 10] and *k* ∈ [8, 20]. Parameters *k*, *u* and *x*_0_ were used as secret keys.

### 2.2. The Confusion Process

In the confusion process, a permutation operation is performed on the pixel level with a position transformation. The operational process consists of the following steps:

Step 1: By using specified parameter values *x*_0_, *u* and *k*, iterate the new chaotic system (*N*_0_ + *L*) times and select the rear *L* elements to make a sub chaotic sequence **X** *=* [*x*(1), *x*(2), …, *x*(*L*)]. Where *N*_0_ is an integer used as a security key. 

Step 2: Sequence **X** is sorted in ascending order. Then, one can obtain a sorted chaotic sequence **SX** = [*sx*(1), *sx*(2), …, *sx*(*L*)] and a permutation position array **X**′ = [*x^′^*(1), *x^′^*(2), …, *x^′^*(*L*)], where *x*^ʹ^(*i*) are integers ranging from 1 to *L*. If *x*(*i*) = *sx*(*j*), then *x^′^*(*i*) = *j*.

Step 3: Get the permuted image pixel sequence **P**′ = [*p^′^*(1), *p^′^*(2), …, *p^′^*(*L*)] by using the permutation position array **X***^′^* and the plain image pixel sequence **P**. The transformation relation is

*p^′^*(*i*) = *p* (*x^′^*(*i*)). (2)

### 2.3. Diffusion Process

In the diffusion process, pixel value encryption is performed based on a diffusion array **D**^ʹ^. The operational process consists of the following two steps:

Step 1: Generate the diffusion array **D**′ **= [***d′*(1), *d′*(2), …, *d′*(*L*)] from the chaotic sequence **X** as:

(3) $$d\prime (i)=\mathrm{mod}(\left\lfloor x(i)\times 10^{k}\right\rfloor ,256).$$

Step 2: Get the temporary ciphered image pixel array **C** = [*c*(1), *c*(2), …, *c*(*L*)] from the diffusion vector **D^′^** and the permuted image array **P***′* according to the following diffusion equation:

(4) $$\left\{ \begin{array}{l} c(i)=\mathrm{mod}(p\prime (i)+d\prime (i),256)⊕seed,\mathrm{if}\;i=1,\\ c(i)=\mathrm{mod}(p\prime (i)+d\prime (i),256)⊕c(i-1),\mathrm{if}\;i>1, \end{array}\right.\;$$

where ⊕ denotes the binary *XOR* operator. *c*(*i* − 1) is the previous cipher pixel, and *seed* is a preset constant.

### 2.4. Linear Transformation

**Figure 2:**
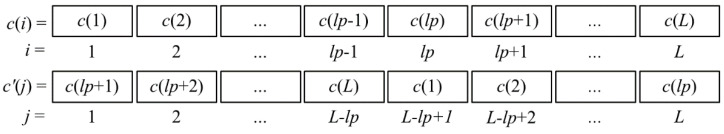
The linear transformation operation.

Get the final cipher image pixel array **C**^ʹ^ = [*c^′^*(1), *c^′^*(2), …, *c^′^*(*L*)] from the temporary cipher image pixel array **C** and a security number *lp* as

(5) $$\left\{ \begin{array}{l} c\prime (i-lp)=c(i),\mathrm{if}\;i>lp,\\ c\prime (i-lp+L)=c(i),\mathrm{if}\;i\leq lp, \end{array}\right.\;$$

where *lp* is used as a security key. In order to see the result of the linear transformation at a glance, we used a graph to express the linear transformation process, which is shown in Figure 2. There are two key points in this linear transformation process, which deserve our special attention. One, the first pixel in the array **C** was moved to the (*L* − *lp* + 1) position in the array **C**ʹ, that is *c^′^*(*L* − *lp* + 1) = *c*(1). Second, the original two adjacent pixels *c*(*lp*) and *c*(*lp* + 1) were moved to the end and start of the array **C**ʹ, that is, *c^′^*(*L*) = *c*(*lp*), *c^′^*(1) = *c*(*lp* + 1). If *lp* = 0 or *lp* = *L*, then *c^′^*(*i*) = *c*(*i*). Hence, a reasonable range of *lp* is 0 < *lp < L.*

The final cipher image was obtained by converting the 1D pixel vector **C***′* into a 2D color image consisting of R, G and B components with the size of *M* × *N*. The secret keys used in Pak’s algorithm consists of five parameters {*x*_0_, *u*, *k*, *N*_0_, *lp*}.

The decryption process is the inverse operation of the encryption process and it was omitted here. 

According to Kerchoff’s principle, when analyzing an encryption algorithm, an assumption is made that the cryptanalyst knows exactly the design and working of the cryptosystem. Namely, the only thing the attacker does not know is the secret key. The definition of a chosen-plaintext attack can be described as follows: Attackers have the chance to use the encryption machine temporarily, hence they can select a special plaintext to encrypt and get its corresponding ciphertext without knowing the secret keys. 

In Pak’s algorithm, the permutation position array **X***′* and the diffusion array **D***′* are determined by parameters {*x*_0_, *u*, *k*, *N_0_*} and have nothing to do with the plain image. Namely, **X***′* and **D***′* are static and do not change with different images to be encrypted. The secret key, *lp*, and the unknown parameter *seed* also have nothing to do with the plain image. Therefore, attackers can choose some special plaintext images to encrypt by using Pak’s encryption machine when they temporarily obtain the opportunity to use Pak’s encryption machine and obtain the corresponding ciphertext image to use these known plaintext-ciphertext image pairs to crack the equivalent key sequences **X***′***, D***′*, parameters *lp* and *seed.* By using these equivalent key sequences **X***′* and **D***′*, parameters *lp* and *seed*, any image encrypted by Pak’s encryption machine can be decrypted without knowing the original keys of Pak’s encryption machine. This is the basic principle of the chosen-plaintext attack model. According to this attack model, it is obvious that Pak’s scheme cannot resist a chosen-plaintext attack. 

### 2.5. The Related Cryptanalysis Work

In Wang’s cryptanalysis scheme, the authors constructed an equivalent cryptosystem for Pak’s cryptosystem. In the equivalent cryptosystem, they constructed the new permutation position array **X***″* and diffusion array **D***″* of the equivalent encryption scheme by transforming the original permutation position array **X***′* and diffusion array **D***′* with the secret parameter *lp* respectively. The relationships of the key streams between the equivalent cryptosystem and Pak’s cryptosystem are as follows

(6) $$\left\{ \begin{array}{l} x\prime \prime (i-lp)=x\prime (i),\;\mathrm{if}\;i\in (lp,L],\\ x\prime \prime (i-lp+L)=x\prime (i),\;\mathrm{if}\;i\in [1,lp]. \end{array}\right.\;$$

(7) $$\left\{ \begin{array}{l} d\prime \prime (i-lp)=d\prime (i),\;\mathrm{if}\;i\in (lp,L],\\ d\prime \prime (i-lp+L)=d\prime (i),\;\mathrm{if}\;i\in [1,lp]. \end{array}\right.\;$$

Wang’s equivalent encryption scheme contains only two processes: permutation and diffusion, which can be described by Equations (8) and (9) respectively.

*p″*(*i*) = *p*(*x″*(*i*)) (8)

(9) c″(i)=mod(p″(i)+d″(i),256)⊕c″(i−1) 

where **P***″* = [*p″*(1), *p″*(2), …, *p″*(*L*)] is the permuted image pixel sequence of Wang’s equivalent cryptosystem, which has the following relations with **P***′* in Pak’s system

(10) $$\left\{ \begin{array}{l} p\prime \prime (i-lp)=p\prime (i),\;\mathrm{if}\;i\in (lp,L],\\ p\prime \prime (i-lp+L)=p\prime (i),\;\mathrm{if}\;i\in [1,lp]. \end{array}\right.\;$$

**C***″* = [*c″*(1), *c″*(2), …, *c″*(*L*)] is the final cipher image pixel array of Wang’s equivalent cryptosystem. The authors claim that c″(i)=c′(i) will hold if the Equations (6)–(10) hold.

The operation process of Wang’s chosen-plaintext attack scheme is divided into the following three stages.

(1) Extract the diffusion array **D***″*. Select a special plain-image **P** consisting of all 0 elements such that *p″*(*i*) = 0 and obtain the corresponding cipher-image **C***″*. According to Equation (9), the diffusion array **D***″* is extracted as

(11) d″(i)=c″(i)⊕c″(i−1).

(2) Extract the permutation position array **X***″*. Select *L* special plain images with the 1D pixel arrays respectively denoted as **P**_1_, **P**_2_, …, **P***_L_* and the *j*th element in the pixel array **P***_j_* is 1; all other elements are 0. Get the corresponding encrypted image arrays **C**_1_, **C**_2_, …, **C***_L_.* By using one plain image **P***_j_* and the corresponding **C***_j_*, only one element *x″*(*i*) in **X**″ can be obtained. All elements of {*x″*(1), *x″*(1), …, *x″*(*L*)} can be obtained when *L* pairs of (**P***_j_*, **C***_j_*) are used. 

(3) Recover the original plain image. By using the new permutation position array **X***″* and the new diffusion array **D***″*, recover the original plain image **P** from the target cipher image **C**. 

We find that Wang’s cryptanalysis algorithm has the following issues: 

(1) The authors assume that the attacker knows the parameter *seed* and used it as a known parameter in the equivalent encryption system. In fact, the *seed* parameter is a constant set in Pak’s cryptosystem. Although the attacker can use Pak’s encryption machine temporarily, the *seed* parameter is unknown to the attacker. 

(2) Although the authors claim that the cipher image **C***″* obtained from their equivalent cryptosystem is the same as the cipher image **C***′* obtained by the original Pak’s cryptosystem, no strict proof is given. In fact, the cipher image pixel array **C***″* is not equivalent to **C***′* due to the unknown parameter *lp*, which is not broken out by the authors. The proof procedure is as follows. 

When encrypting the first pixel by Wang’s equivalent cryptosystem, Equation (9) is degenerated into the form as *c″*(1) = mod(*p″*(1) + *d″*(1), 256)⊕*c″*(*0*)**,** where c″(0) is not a pixel value of the array **C***″* and c″(0) may be the parameter *seed*. From Equations (7), (9) and (10), we can get *p″*(1) = *p′*(*lp* + 1) and *d″*(1) = *d′*(*lp* + 1). Then one can obtain *c″*(1) as

*c″*(1) = mod(*p′*(*lp* + 1)+*d′*(*lp* + 1), 256)⊕*seed.* (12)

while c′(1) obtained by using Pak’s algorithm is as

*c′*(1)= mod(*p′*(*lp* + 1)+*d′*(*lp* + 1), 256)⊕*c′*(*L*). (13)

By comparing Equations (12) with (13), *c″*(1) ≠ *c′*(1). 

When *i* = *L* – *lp* + 1, Equation (9) is degenerated into the form *c″*(*L* – *lp* + 1)=mod(*p″*(*L* – *lp* + 1) + *d″*(*L* – *lp* + 1), 256)⊕*c″*(*L* − *lp*). From Equations (7), (9) and (10), we can get *p″*(*L* – *lp* + 1) = *p′*(1) and *d″*(*L* – *lp* + 1) = *d′*(1). As a result, *c″*(*L* – *lp* + 1) is as

*c″*(*L* − *lp* + 1) = mod(*p′*(1) + *d′*(1), 256)⊕*c″*(*L* − *lp*). (14)

while using Pak’s algorithm, *c′*(*L* – *lp* + 1) is as

*c′*(*L* − *lp* + 1) = mod(*p′*(1) + *d′*(1), 256)⊕*seed.* (15)

Comparing Equations (14) with (15), *c″*(*L* – *lp* + 1) ≠ *c′*(*L* – *lp* + 1). 

Based on *c″*(1) ≠ *c′*(1)**,** one can deduce that *c″*(*i*) ≠ *c′*(*i*), *i* = 2, 3, …, *L*.

In fact, there are some defects in Wang’s cryptanalysis algorithm because the authors completely ignore the role of the parameter *lp* and do not break out *lp*. However, when the parameter *lp* is not known, one cannot know where the *seed* should be used to calculate *c″*(*i*). 

(3) The most serious problem in Wang’s cryptanalysis scheme is that the number of chosen plain images is too high to reach *M* × *N* × 3 in extracting the permutation position array **X***″*. The use of one chosen plain image at a time can only break one element value of **X***″*, which is very inefficient, so Wang’s cryptanalysis scheme is unrealistic.

In Chen’s cryptanalysis scheme, unfortunately, the parameter *seed* is also not deciphered and used as a known parameter. Thus, reducing the difficulty of the cryptanalysis. In addition, Chen did not give the specific process of deciphering the permutation position array **X***′*.

## 3. The Improved Cryptanalysis Scheme

In order to provide a more comprehensive and efficient cryptanalysis method on Pak’s encryption algorithm, we propose an improved chosen-plaintext attack algorithm to Pak’s scheme. Suppose the target color cipher image to be decrypted has the size of *L* = M×N×3. Firstly, we cracked the secret parameter *lp* and the diffusion array [*d′*(2), *d′*(3), …, *d′*(*L*)] except for *d′*(1) by using two selected plain images and their corresponding cipher images. Secondly, we cracked the unknown parameter *seed* and *d′*(1) by using one or more than one selected plain images. Thirdly, we cracked the permutation position array **X***′* by using ⌈(M×N×3)/255⌉ selected plain images and their corresponding cipher images, where ⌈x⌉ is the smallest integer that is greater than or equal to *x*. Wang’s cryptanalysis algorithm needs M×N×3 selected plain images to decipher the permutation position array **X***′*, while our cryptanalysis algorithm only needs ⌈(M×N×3)/255⌉ selected plain images to decipher the permutation position array **X***′*. Hence, the efficiency of our improved chosen-plaintext attack algorithm is about 255 times that of Wang’s algorithm. 

### 3.1. Recover the Secret Key lp and the Diffusion Array

**Figure 3:**
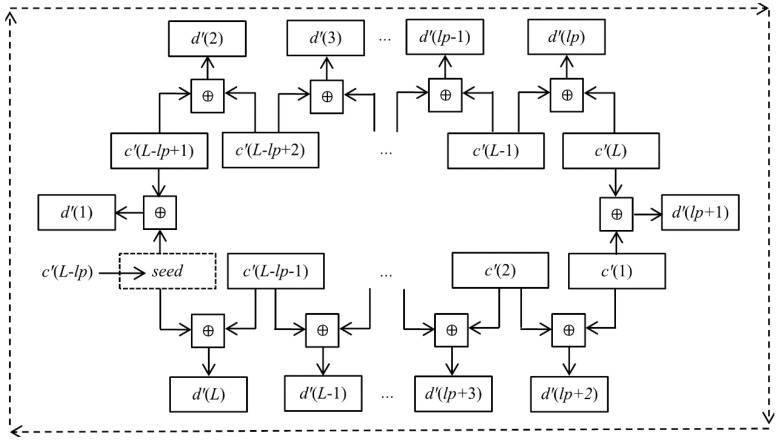
The diagram of the relationship between **D***′* and **C***′*.

According to Equations (4) and (5), d′(i) can be calculated as

(16) $$\left\{ \begin{array}{l} d\prime (1)=c\prime (L-lp+1)⊕seed\dot{-}p\prime (1),\mathrm{if}\;i=1,\\ d\prime (i)=c\prime (L-lp+i)⊕c\prime (L-lp+i-1)\dot{-}p\prime (i),\mathrm{if}\;1<i\leq lp,\\ d\prime (lp+1)=c\prime (1)⊕c\prime (L)\dot{-}p\prime (lp+1),\mathrm{if}\;i=lp+1,\\ d\prime (i)=c\prime (i-lp)⊕c\prime (i-lp-1)\dot{-}p\prime (i),\mathrm{if}\;lp+1<i\leq L, \end{array}\right.\;$$

where *x*$\dot{-}$*y* = mod(*x* – *y* + 256, 256). Obviously, if the *seed* in Equation (16) is replaced by *c′*(*L − lp*), then the relationship between *d′*(*i*) and *c′*(*j*) can be expressed in Figure 3.

From Figure 3, one can see that each key *d′*(*i*) is related to a pair of adjacent pixel values {*c′*(*j*), *c′*(*j* + 1)} or {*c′*(*L*), *c′*(1)}. To avoid the influence of the unknown parameter *lp*, we can select a specific plain image where all pixels *p′*(*i*) have the same value *q*, then we can calculate a series of values by neighbors {*c′*(*L*), *c′*(1)}, {*c′*(1), *c′*(2)}, {*c′*(2), *c′*(3)}, …, {*c′*(*L* − 1), *c′*(*L*)} and store these values in a temporary array **D** = [*d*(1), *d*(2), …, *d*(*L*)], where *d*(*i*) is as

(17) $$\left\{ \begin{array}{l} d(1)=c\prime (1)⊕c\prime (L)\dot{-}q,\\ d(i)=c\prime (i)⊕c\prime (i-1)\dot{-}q,\;i=2,3,\ldots ,L. \end{array}\right.\;$$

Equation (17) brings us great convenience for computing *d*(*i*) because it does not contain the unknown parameters *lp* and *seed*. Obviously, the equivalent relationship of the elements between **D**′ = [*d′*(1), *d′*(2), …, *d′*(*L*)] and **D** = [*d*(1), *d*(2), …, *d*(*L*)] is as follows

(18) $$\left\{ \begin{array}{l} d\prime (i)=d(i-lp),\mathrm{if}\;i>lp,\\ d\prime (i)=d(L+i-lp),\mathrm{if}\;i\leq lp. \end{array}\right.\;$$

Namely, *d′*(*lp* + 1) = *d*(1), *d′*(*lp* + 2) = *d*(2), …, *d′*(*L* − 1) = *d*(*L* – *lp* − 1), *d′*(*L*) = *d*(*L* − *lp*); *d′*(1) = *d*(*L* – *lp +* 1), *d′*(2) = *d*(*L* – *lp* + 2), …, *d′*(*lp* − 1) = *d*(*L* − 1), *d′*(*lp*) = *d*(*L*).

It is worth noting that, except for *d′*(1) or *d*(*L* – *lp* + 1), the rest of the values *d*(*i*) (*i* ≠ *L* – *lp* + 1) obtained by Equation (17) are all right values. Namely, when calculating *d*(*L – lp +* 1), if we do not use the parameter *seed* and use the *c′*(*L − lp*) value instead of *seed*, then the result of *d*(*L* – *lp +* 1) may be wrong. Considering the values of *d*(*i*) or *d′*(*i*) are determined by parameters {*x*_0_, *u*, *k*, *N*_0_} and have nothing to do with the content of the image, if we choose two different plain images and get the corresponding cipher images, by using the two pairs of plaintext-ciphertext to calculate *d*_1_(*i*) and *d*_2_(*i*), then one can find the only position of *ii* that the value of *d*_1_(*ii*) and *d*_2_(*ii*) will not be identical but values of *d*_1_(*i*) and *d*_2_(*i*) at other locations *i* (*i* ≠ *ii*) are definitely the same. Once the location *ii* is sought out, the value of *lp* can be determined, which is *lp* = *L* + 1 − *ii*.

Based on the above idea, we get the algorithm for deciphering the secret key parameter *lp* and the diffusion array *d′*(*i*), which is described as follows: 

Step 1: Let *q* = 0, and select a special plain image **PA** = [*pa*(1), *pa*(2),…, *pa*(*L*)] that all pixels *pa*(*i*) have the same value *q* and obtain the corresponding cipher image **CA**′ = [*ca′*(1), *ca′*(2), …, *ca′*(*L*)] by using Pak’s encryption machinery. As **PA**′ = [*q*, *q*, …, *q*], then we can get a array **DA** = [*da*(1), *da*(2), …, *da*(*L*)] by using Equation (17).

Step 2: Let *q* = *q* + 1, and select a special plain image **PB** = [*pb*(1), *pb*(2), …, *pb*(*L*)] that all pixels *pb*(*i*) have the same value *q*. Obtain the corresponding cipher-image **CB**′ = [*cb′*(1), *cb′*(2), …, *cb′*(*L*)] by using Pak’s encryption machine. Because **PB**′ = [*q*, *q*, …, *q*], then we can get another array **DB** = [*db*(1), *db*(2), …, *db*(*L*)] by using Equation (17).

Step 3: Compare *da*(*i*) and *db*(*i*) one by one for *i* = 1, 2, …, *L*. If it exists at position *I* = *ii* and meets the relationship *da*(*ii*) ≠ *db*(*ii*), then *L – lp +* 1 = *ii*, so *lp* is determined as *lp* = *L* + 1 − *ii*, and go to Step 4. Otherwise, repeat Step 2 to Step 3 until *lp* is determined.

Step 4: After the value of *lp* is ascertained, we can recover the diffusion array **D**’ of Pak’s cryptosystem by using Equation (18). Where only the value of *d′*(1) is incorrect.

### 3.2. Recover d′(1) and the Unknown Parameter Seed

According to the first formula in Equation (16), (*d′*(1), *seed*) meets the following relationship

(19) c′(L−lp+1)=mod(d′(1)+p′(1),256)⊕seed.

Using the special chosen plain image **PA** = [0, 0, …, 0] and **PB**= [1, 1, …, 1], we have got a pair of ciphertext data (*ca′*(*L* – *lp* + 1), *cb′*(*L* – *lp* + 1)) in the previous section. Therefore, *d′*(1) and *seed* needs to satisfy the following equation:

(20) $$\{\begin{array}{c} ca\prime (L-lp+1)=\mathrm{mod}(d\prime (1)+0,256)⊕seed,\\ cb\prime (L-lp+1)=\mathrm{mod}(d\prime (1)+1,256)⊕seed. \end{array}\;$$

Consider such a fact that *seed* ∈ {0, 1, 2, …, 255} and *d′*(1) ∈ {0, 1, 2, …, 255}, so the solution of Equation (20) can be easily obtained by the computer exhaustive algorithm. However, the solution [*d′*(1), *seed*] of Equation (20) is not unique because the equations in Equation (20) are not two linear equations. Suppose an equation for *d′* and *seed* has the following form:

mod(*d′* + *q*, 256)⊕*seed = c′.* (21)

Regarding the solutions of Equation (21), We have the following Proposition:

**Proposition** **1.** *For any values of q ∈ Z_256_ and c′ ∈ Z_256_, if [d′, seed] is a solution of Equation (21), then [mod(d′ + 128, 256), mod(seed + 128, 256)] is also a solution of Equation (21). Where d′ ∈ Z_256_ and seed ∈ Z_256_.*

**Proof.**  Suppose the binary value of mod(*d′ + q*, 256) is ${\left(d_{8}d_{7}d_{6}d_{5}d_{4}d_{3}d_{2}d_{1}\right)}_{2}$ and the binary value of *seed* is ${\left(s_{8}s_{7}s_{6}s_{5}s_{4}s_{3}s_{2}s_{1}\right)}_{2}$.

If *d*_8_ = 0, then mod(mod(*d′ +* 128, 256) *+ q*, 256) = mod(*d′ + q +* 128, 256) = mod(mod(*d′ + q*, 256) + 128, 256) = mod((0*d*_7_*d*_6_*d*_5_*d*_4_*d*_3_*d*_2_*d*_1_)_2_ + (10000000)_2_, 256) = (1*d*_7_*d*_6_*d*_5_*d*_4_*d*_3_*d*_2_*d*_1_)_2_ = ${\left({\bar{d}}_{8}d_{7}d_{6}d_{5}d_{4}d_{3}d_{2}d_{1}\right)}_{2}$. Where, $\bar{x}$ represents the binary inverse value of *x*.

If *d*_8_ = 1, then mod(mod(*d′ +* 128, 256) *+ q*, 256) = mod(*d′ + q +* 128, 256) = mod(mod(*d′ + q*, 256) + 128, 256) = mod((1*d*_7_*d*_6_*d*_5_*d*_4_*d*_3_*d*_2_*d*_1_)_2_ + (10000000)_2_, 256) = (0*d*_7_*d*_6_*d*_5_*d*_4_*d*_3_*d*_2_*d*_1_)_2_ = ${\left({\bar{d}}_{8}d_{7}d_{6}d_{5}d_{4}d_{3}d_{2}d_{1}\right)}_{2}$.

If *s*_8_ = 0, then mod(*seed +* 128, 256) = mod(${\left(0s_{7}s_{6}s_{5}s_{4}s_{3}s_{2}s_{1}\right)}_{2}$ + (10000000)_2_, 256) =${\left(1s_{7}s_{6}s_{5}s_{4}s_{3}s_{2}s_{1}\right)}_{2}$ = ${\left({\bar{s}}_{8}s_{7}s_{6}s_{5}s_{4}s_{3}s_{2}s_{1}\right)}_{2}$.

If *s*_8_ = 1, then mod(*seed +* 128, 256) = mod(${\left(1s_{7}s_{6}s_{5}s_{4}s_{3}s_{2}s_{1}\right)}_{2}$ + (10000000)_2_, 256) =${\left(0s_{7}s_{6}s_{5}s_{4}s_{3}s_{2}s_{1}\right)}_{2}$ = ${\left({\bar{s}}_{8}s_{7}s_{6}s_{5}s_{4}s_{3}s_{2}s_{1}\right)}_{2}$. □

Considering ${\bar{d}}_{8}⊕{\bar{s}}_{8}=d_{8}⊕s_{8}$, we can obtain that mod(mod(*d′ +* 128, 256) *+ q*, 256) ⊕ mod(*seed +* 128, 256) = ${\left({\bar{d}}_{8}d_{7}d_{6}d_{5}d_{4}d_{3}d_{2}d_{1}\right)}_{2}$⊕ ${\left({\bar{s}}_{8}s_{7}s_{6}s_{5}s_{4}s_{3}s_{2}s_{1}\right)}_{2}$=(*d*_8_*d*_7_*d*_6_*d*_5_*d*_4_*d*_3_*d*_2_*d*_1_)_2_⊕${\left(s_{8}s_{7}s_{6}s_{5}s_{4}s_{3}s_{2}s_{1}\right)}_{2}$= *c′.* This means that [mod(*d′ +* 128, 256), mod(*seed +* 128, 256)] is also a solution of Equation (21).

Suppose Equation (20) has *m* groups of solutions (*m* ≥ 2) as [*d*_1_*′*(1), *seed*_1_], [*d*_2_*′*(1), *seed*_2_],…, [*d_m_′*(1), *seed_m_*]. If *m* = 2, then the two groups of solutions are all the required results and the task of recovering (*d′*(1), *seed*) has been completed. If *m* > 2, then we must select some other plain image **P** = [*q*, *q*, …, *q*] and obtain the corresponding cipher image **C**′ = [*c′*(1), *c′*(2), …, *c′*(*L*)], where *q* > 1. In addition, we can obtain another equation as: mod(*d′*(1) + *q*, 256) ⊕ *seed = c′*(*L* – *lp* + 1). Under the constraint of the other equation, we can remove those superfluous solutions that do not satisfy all equations until the remaining solutions are only 2 groups. In this way, the unknown parameter *seed* and the secret key *d′*(1) of the original encryption system can be deciphered. The concrete algorithm for recovering *d′*(1) and *seed* is described as follows:

Step 1: Let *m* groups of solutions of Equation (20) be saved in the array **R** = [*r*(1), *r*(2),…, *r*(*m*)] and **S** = [*s*(1), *s*(2),…, *s*(*m*)] sequentially, Where *r*(*i*) = *d_i_′*(1), *s*(*i*) = *seed_i_*, *i* = 1, 2, …, *m.* Let *q* = 1.

Step 2: Check the value of *m*. If *m* ≤ 2, then go to Step 9. If *m* > 2, then go to Step 3.

Step 3: *q* = *q* + 1.

Step 4: For *i* = 1, 2, …, *m*, each groups of solutions [*r*(*i*), *s*(*i*)] is assumed to be used to encrypt the plaintext pixel value *q* and calculate the corresponding ciphertext values as *cc*(*i*) = mod(*q* + *r*(*i*), 256)⊕*s*(*i*).

Step 5: For *i* = 1, 2, …, *m*, Check whether the value of each element in the array [*cc*(1), *cc*(2), …, *cc*(*m*)] is exactly the same. If *cc*(*i*) is exactly the same, then repeat Step 3 to Step 5. If *cc*(*i*) is not exactly the same, then go to Step 6.

Step 6: Select a special plain image array **P** = [*q*, *q*, …, *q*] and obtain the corresponding cipher image pixels array **C***′* = [*c′*(1), *c′*(2), …, *c′*(*L*)] by using Pak’s encryption machine. 

Step 7: For each solution group [*r*(*i*), *s*(*i*)], calculate the values of mod(*r*(*i*) + *q*, 256)⊕*s*(*i*), *i* = 1, 2,…, *m*. If mod(*r*(*i*) + *q*, 256)⊕*s*(*i*) ≠ *c′*(*L* – *lp* + 1), then delete the *i*-th solution group [*r*(*i*), *s*(*i*)] from **S** and **R** respectively. 

Step 8: Modify the value of *m*, that is, *m* = size(**R**), and return to Step 2.

Step 9: Output the final values of [*d′*(1), *seed*], that is [*d′*(1), *seed*] = [*r*(1), *s*(1)] or [*d′*(1), *seed*] = [*r*(2), *s*(2)]. 

### 3.3. Recover the Permutation Position Array **X**′

**Figure 4:**
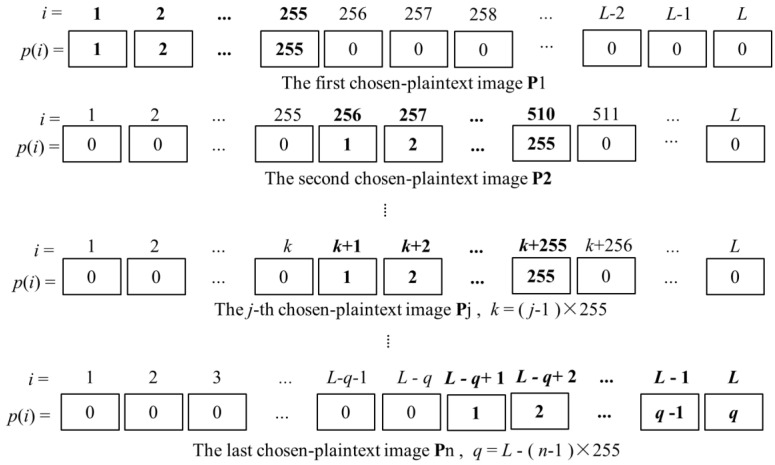
The patterns of elements in each chosen plain image pixel array **P***_j_*.

After the RGB image matrix is converted into a 1D gray image pixel sequence **P** = [*p*(1), *p*(2), …, *p*(*L*)], array **P** has *L* pixels and *L* = *M* × *N* × 3. Each value of *p*(*i*) is an integer in the range of [0, 255]. If *L* ≤ 255, then only one chosen-plain image **P** = [1, 2, …, *L*] is necessary to recover the permutation position array **X***′*, so that each pixel in the chosen plain image has different values in {1, 2, …, *L*}. If *L* > 255, then *n* chosen plain images are required to recover the permutation position array **X***′*, where *n* = ⌈L/255⌉ > 1. In this case, we select a series of special color plain images (**P**_1_, **P**_2_, …, **P***_n_*) and **P***_j_* = [*p_j_*(1), *p_j_*(2), …, *p_j_*(*L*)]. We divide **P***_j_* into *n* groups and each group contains 255 pixels except for the last one and the last group contains *q* pixels (*q* ≤ 255). For the *j*-th chosen-plain image pixel array **P***_j_*, we assign each element of the *j*-th group a distinct value between 1 to 255 and the others are assigned the value of 0. The patterns of elements in each chosen plain image pixel array **P***_j_* are shown in Figure 4. 

We then obtain the corresponding series of cipher images ($C_{1}\prime$, $C_{2}\prime$, …, $C_{n}\prime$) by using Pak’s encryption machine. Where, $C_{j}\prime$ = [$c_{j}\prime (1)$, $c_{j}\prime (2)$, …, $c_{j}\prime (L)$]. Then, we can decrypt $C_{j}\prime$ to obtain $P_{j}\prime$ = [$p_{j}\prime (1)$, $p_{j}\prime (2)$, …, $p_{j}\prime (L)$], where $p_{j}\prime (i)$ can be obtained by using Equation (16).

Finally, because of the relationship *p*_j_′(*i*) = *p_j_*(*x*′(*i*)) (*i* ∈ [1, *L*]), **X***′* can be determined by comparing $P_{j}\prime$ and $P_{j}$. Namely, if $p_{j}\prime (i)=p_{j}(k)$, then *x*′(*i*) = *k*.

### 3.4. Recover the Original Plain Image

In Section 3.1 to 3.3, we obtained the secret keys {*lp*, **X***′*, **D***′*} and the unknown parameter *seed*, which are unrelated to the plain image or ciphertext image. Therefore, we can decrypt any other ciphertext image **CI** by using the parameter set {*seed*, *lp*, **X***′*, **D***′*}. The decryption process to recover the plain image **PI** from the target ciphertext image **CI** is exactly the same as the decryption process of Pak’s scheme, which can be described as follows: 

Step 1: Convert the color ciphertext image **CI** with a size of *M* × *N* × 3 into a 1D pixel array **C***′* = [*c′*(1), *c′*(2), …, *c′*(*L*)], where *L*= *M* × *N* × 3.

Step 2: Obtain the intermediary cipher image pixel array **C** = [*c*(1), *c*(2), …, *c*(*L*)] from the final cipher pixel array **C***′* = [*c′*(1), *c′*(2), …, *c′*(*L*)] by performing the inverse transformation of Equation (5).

Step 3: Recover the permuted image pixel array **P***′* = [*p′*(1), *p′*(2), …, *p′*(*L*)] by performing the inverse diffusion process of Equation (4).

Step 4: Do inverse permutation on **P***′* to obtain **P** by using the inverse permutation process of Equation (2).

Step 5: Convert the 1D array **P** into a 3D matrix with a size of *M* × *N* × 3 and the original color plain image **PI** is recovered.

### 3.5. Examples of the Improved Cryptanalysis Scheme

Suppose the right values of original secret keys in Pak’s cryptosystem are as follows: *x*_0_ = 0.456, *u* = 5.4321, *k* = 14, *N*_0_ = 1000, *lp* = 5, and *seed* = 250.

**Figure 5:**
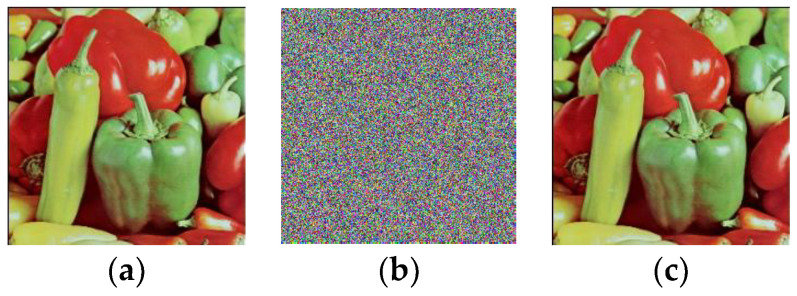
The experimental results of the chosen-plaintext attacks. (**a**) The plain image; (**b**) the cipher image; (**c**) the cracked image.

**Example** **1.** *In this example, the plain image **P** is the color peppers with a size of 256 × 256 × 3. The plain image and its cipher image encrypted by using Pak’s encryption machine are shown in Figure 5a,b respectively. The deciphered image by using our chosen-plaintext attack is shown in Figure 5c, which is exactly the same as the original plain image in Figure 5a. Through the image peppers as an example, our attack attains demonstration.*

**Example** **2.** *The secret key parameters are the same as those of Example 1. In order to verify the correctness of our chosen-plaintext attack scheme more intuitively, this example shows a simple and specific numerical experiment. In this example, the plain image **PI** is the color image with size of 2 × 2 × 3 (L = M × N × 3 = 12), and its components are as*

(22) $$P_{R}=\left[\begin{array}{cc} 11 & 13\\ 12 & 14 \end{array}\right],P_{G}=\left[\begin{array}{cc} 21 & 23\\ 22 & 24 \end{array}\right],P_{B}=\left[\begin{array}{cc} 31 & 33\\ 32 & 34 \end{array}\right].$$

Its corresponding 1D pixel array **P** is:

**P** = [11, 12, 13, 14, 21, 22, 23, 24, 31, 32, 33, 34]. (23)

As the result, the 1D pixel array **C***′* encrypted by Pak’s encryption machine is:

C*′* = [246, 16, 1, 6, 37, 3, 137, 197, 162, 215, 51, 22]. (24)

By choosing two special plain-image array **PA** = [0, 0, 0, 0, 0, 0, 0, 0, 0, 0, 0, 0] and **PB** = [1, 1, 1, 1, 1, 1, 1, 1, 1, 1, 1, 1], we obtain the corresponding cipher image arrays as **CA***′* = [173, 117, 137, 108, 97, 110, 17, 229, 170, 195, 20, 18] and **CB***′* = [255, 38, 219, 61, 51, 35, 163, 218, 138, 224, 56, 63]. According to Equation (16), we obtain **DA** and **DB** as: **DA** = [191, 216, 252, 229, 13, 15, 127, **244**, 79, 105, 215, 6], **DB** = [191, 216, 252, 229, 13, 15, 127, **120**, 79, 105, 215, 6]. By comparing **DA** and **DB**, we find that *da*(8) ≠ *db*(8), then *ii* = 8, and *lp = L +* 1 – *ii* = 12 + 1 – 8 = 5. Then, Equation (19) is changed into the following form

$$\{\begin{array}{c} ca\prime (8)=229=\mathrm{mod}(d\prime (1)+0,256)⊕seed\\ cb\prime (8)=218=\mathrm{mod}(d\prime (1)+1,256)⊕seed \end{array},$$

which has four groups of solution: [*d′*(1), *seed*] = {[31, 250], [95, 186], [159, 122], [223, 58]}. For *q* = 2, 3, …, check the values of “mod(*d′*(1) + *q*, 256)⊕*seed*” with the four groups of solution. When *q* = 33, we find that “mod(*d′*(1) + *q*, 256)⊕*seed*” has different values (186, 58, 186, 58) corresponding to the four groups of solution. We then select a special color plain image **P =** [33, 33, …, 33] and obtain the corresponding cipher image **C***′* = [127, 134, 155, 157, 179, 131, 35, **186**, 202, 64, 184, 159] by using Pak’s encryption machine, in which *c′*(8) = 186. Then we can determine that (31, 250) and (159, 122) are two right groups of secret keys to [*d′*(1), *seed*]. If we adopt [*d′*(1), *seed*] = [159, 122] as the secret keys, then we can obtain **D***′* from **DA** or **DB** by using Equation (18), that is, **D***′* = [**159**, 79, 105, 215, 6, 191, 216, 252, 229, 13, 15, 127].

To recover the permutation position array **X***′*, we select a special color plain image **P =** [1, 2, 3, 4, 5, 6, 7, 8, 9, 10, 11, 12] and obtain the corresponding cipher image **C***′* = [240, 44, 45, 220, 207, 217, 89, **211**, **132, 239, 53, 58**] by using Pak’s encryption machine. Then we can obtain its intermediary ciphertext array **C** according to Equation (5) as **C** = [**211**, **132, 239, 53, 58**, 240, 44, 45, 220, 207, 217, 89]. Then we can obtain the permutated pixel array **P***′* from **D***′* by using Equation (4), that is, **P***′* = [10, 8, 2, 3, 9, 11, 4, 5, 12, 6, 7, 1]. By comparing **P** and **P***′*, the permutation array **X***′* is recovered as **X***′* = [10, 8, 2, 3, 9, 11, 4, 5, 12, 6, 7, 1].

For the target ciphertext array C*′* of Equation (24), we obtain its intermediary cipher pixel array **C** according to Equation (5) as **C** = [197, 162, 215, 51, **22**, 246, 16, 1, 6, 37, 3, 137]. Then we obtain the permutated pixel array **P***′* from **D***′* by using Equation (4), that is, **P***′* = [32, 24, 12, 13, 31, 33, 14, 21, 34, 22, 23, 11]. Finally, according to **X***′*, **P** is recovered as **P** = [11, 12, 13, 14, 21, 22, 23, 24, 31, 32, 33, 34], which coincides with the original plain image array of Equation (23).

Through the two examples, our attack attains demonstration. Therefore, Pak’s encryption scheme cannot resist the chosen-plaintext attacks and the security of the algorithm is not high enough.

## 4. The Improved Cryptosystem

In Pak’s encryption scheme, the diffusion array **D***′* and the permutation position array **X***′* are used separately in the diffusion and permutation stage. Accordingly, the diffusion array **D***′* and the permutation position array **X***′* are easily deciphered separately by the attackers. This is a weakness of Pak’s encryption scheme. In Wang’s improved encryption scheme, a parameter *E* determined by the plaintext image is introduced. In order to obtain the value of the *E* parameter, it is necessary to calculate the average value of all the pixels of the image, which obviously increases the time overhead of the algorithm. In addition, the linear transformation operation of Wang’s algorithm is changed to the binary shift operation to each pixel, which makes encryption speed very slow. 

Our improved algorithm retains the advantages of the speed of the original algorithm and overcomes its shortcomings. It includes two rounds of synchronous operations of diffusion and confusion. Two diffusion arrays **D***′* and **D** are generated by using the chaotic sequence **X** and the previously encrypted pixel value. **D***′* and **D** are used to encrypt the image pixels respectively in the two rounds of synchronous operation.

### 4.1. Encryption Process 

Step 1: Input the secret parameters {*x*_0_, *u*, *k*, *N*_0_, *C*_0_} and the color image **PI** with the size of *M* × *N* × 3, and **PI** is reshaped to a one-dimensional grayscale image array **P** = [*p*(1), *p*(2), …, *p*(*L*)], where *L* = *M* × *N* × 3.

Step 2: By using the parameters of {*x*_0_, *u*, *k*, *N*_0_ }, iterate the new chaotic Sine-Sine system (*L* + *N*_0_) times and abandon the front *N*_0_ elements to make the chaotic sequence **X** = [*x*(1), *x*(2), …, *x*(*L*)].

Step 3: Get the permutation position matrix **X***′* = [*x*′(1), *x*′(2), …, *x*′(*L*)] by sorting the chaotic sequence **X** in ascending order. Where, *x*′(*i*) are integers ranging from 1 to *L*, *i* = 1, 2, …, *L*.

Step 4: Perform the permutation and diffusion operations on array **P** simultaneously and obtain the temporary cipher image pixel array **C***′* = [*c′*(1), *c′*(2), …, *c′*(*L*)] as

(25) $$\left\{ \begin{array}{l} d\prime (1)=\mathrm{mod}(\mathrm{floor}(\frac{x(1)+C_{0}/256}{2}\times 10^{10}),256)\\ c\prime (1)=\mathrm{mod}(p(x\prime (1))+d\prime (1)+C_{0},256) \end{array}\right.\;$$

(26) $$\left\{ \begin{array}{l} d\prime (i)=\mathrm{mod}(\mathrm{floor}(\frac{x(i)+c\prime (i-1)/256}{2}\times 10^{10}),256),\\ c\prime (i)=\mathrm{mod}(p(x\prime (i))+d\prime (i)+c\prime (i-1),256),i>1. \end{array}\right.\;$$

where, **D***′* = [*d′*(1), *d′*(2), …, *d′*(*L*)] is the first diffusion array.

Step 5: Obtain the final cipher image pixel array **C** = [*c*(1), *c*(2), …, *c*(*L*)] from the second diffusion array **D**, permutation position matrix **X***′* and the temporary cipher image pixel array **C***′* as

(27) $$\left\{ \begin{array}{l} d(1)=\mathrm{mod}(\mathrm{floor}(\frac{x(1)+c\prime (L)/256}{2}\times 10^{10}),256)\\ c(1)=\mathrm{mod}(c\prime (1)+d(1)+x\prime (1)+c\prime (L),256) \end{array}\right.\;$$

(28) $$\left\{ \begin{array}{l} d(i)=\mathrm{mod}(\mathrm{floor}(\frac{x(i)+c(i-1)/256}{2}\times 10^{10}),256),\\ c(i)=\mathrm{mod}(c\prime (i)+d(i)+x\prime (i)+c(i-1),256),i>1. \end{array}\right.\;$$

where, **D** = [*d*(1), *d*(2), …, *d*(*L*)] is the second diffusion array.

Step 6: Transform the 1D vector **C** into a 3D matrix with a size of *M* × *N* × 3, then the ciphered color image **CI** is obtained.

### 4.2. Decryption Process

To decrypt the cipher image **CI** with the secret keys {*x*_0_, *u*, *k*, *N*_0_, *C*_0_}, the following decryption operations can be executed. 

Step 1: Transform the 3D matrix **CI** into a gray scale image pixel sequence **C**. 

Step 2: Similar to Step 2 of the encryption process, generate the chaotic sequence **X** = [*x*(1), *x*(2), …, *x*(*L*)]. 

Step 3: Similar to Step 3 of the encryption process, get the permutation position matrix **X**′ = [*x*′(1), *x*′(2), …, *x*′(*L*)] by sorting **X**.

Step 4: Obtain the temporary cipher image pixel array **C***′* = [*c′*(1), *c′*(2), …, *c′*(*L*)] as

(29) $$\left\{ \begin{array}{l} d(i)=\mathrm{mod}(\mathrm{floor}(\frac{x(i)+c(i-1)/256}{2}\times 10^{10}),256),\\ c\prime (i)=\mathrm{mod}(c(i)-d(i)-x\prime (i)-c(i-1),256),i>1. \end{array}\right.\;$$

(30) $$\left\{ \begin{array}{l} d(1)=\mathrm{mod}(\mathrm{floor}(\frac{x(1)+c\prime (L)/256}{2}\times 10^{10}),256)\\ c\prime (1)=\mathrm{mod}(c(1)-d(1)-x\prime (1)-c\prime (L),256) \end{array}\right..$$

Step 5: Obtain the recovered plain image pixel array **P** = [*p*(1), *p*(2), …, *p*(*L*)] as

(31) $$\left\{ \begin{array}{l} d\prime (1)=\mathrm{mod}(\mathrm{floor}(\frac{x(1)+C_{0}/256}{2}\times 10^{10}),256)\\ p(x\prime (1))=\mathrm{mod}(c\prime (1)-d\prime (1)-C_{0},256) \end{array}\right.\;$$

(32) $$\left\{ \begin{array}{l} d\prime (i)=\mathrm{mod}(\mathrm{floor}(\frac{x(i)+c\prime (i-1)/256}{2}\times 10^{10}),256),\\ p(x\prime (i))=\mathrm{mod}(c\prime (i)-d\prime (i)-c\prime (i-1),256),i>1. \end{array}\right.\;$$

Step 6: Transform **P** into a 3D matrix, and the decrypted color image **PI** is obtained. 

## 5. Tests and Analysis for the Improved Cryptosystem

**Figure 6:**
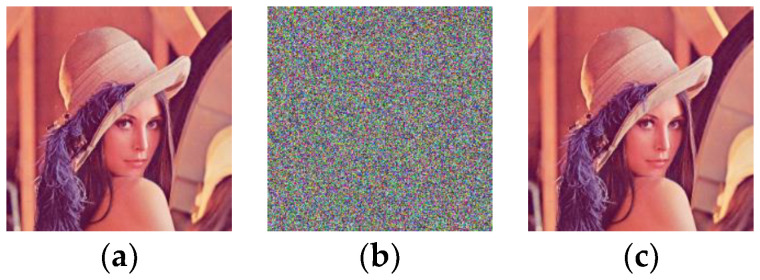
The encryption and decryption effect of the improved scheme. (**a**) The plain image; (**b**) the cipher image; (**c**) the decrypted image.

To examine the performance of the improved cryptosystem, we carried out a simulation experiment. The secret keys were set as (*x*_0_ *=* 0.4563, *u =* 5.4321, *k =* 14, *N*_0_ *=* 1000, *C*_0_ *=* 98). The encryption and decryption algorithms were run on the platform Matlab R2016b in a computer with 3.3 GHz CPU, 4 GB memory and a 64 bit Microsoft Windows 7 operating system. The plain image used in the experiments was the color image lena. Figure 6 shows the original plain image and its cipher image encrypted by the improved scheme. The results reveal that the improved scheme has reliable encryption and decryption effect. 

### 5.1. Resistance to Chosen-Plaintext Attacks

In our improved scheme, the diffusion matrices **D***′* and **D** are related to the temporary and final ciphertext image, which is evident from Equations (25)–(28). It means that images with different contents are encrypted with different diffusion matrices. Furthermore, by using two rounds of diffusion processes, the change of the pixel value at any position in the image will affect all cipher pixel values. Even if the opponent cracked the key streams **D***′* and **D** with some specially selected plain images, the key streams **D***′* and **D** cannot be used to decrypt the target cipher image because the key streams of the target cipher image are different from the cracked key streams. Moreover, it is difficult to decipher the key streams **D***′* and **D** directly by using chosen-plaintext attacks. Therefore, the improved scheme can well resist the chosen-plaintext attacks.

### 5.2. Key Space Analyses

In order to resist a brute-force attack, a cryptographic system must have enough large key space. In our improved cryptosystem, the secret keys include: *x*_0_, *u*, *k*, *N*_0_, *C*_0_, so its key space is 2^128^, which is the same as those in Reference [60]. Under the current computing power, the key space is large enough to resist a brute-force attack. The size of the key space depends not only on the number of keys but also on the number of possible values for each key. The problem of numerical chaotic systems is that the finite precision of the machines (e.g., computers) leads to performance degradation [63,64,65,66], such as the key space is reduced, some weak keys appear, and the randomness of the sequence is reduced. In order to identify and avoid weak keys, we need to calculate the Lyaponuv exponents of chaotic systems or plot the phase space trajectories of the system. 

### 5.3. Statistical Analysis 

#### 5.3.1. Histogram Analysis

**Figure 7:**
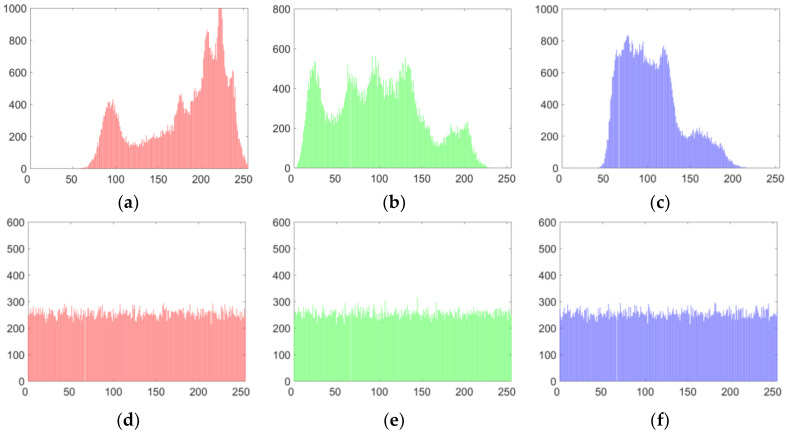
Encryption results for lena. (**a**) The histograms of R component of Figure 6a; (**b**) the histograms of G component of Figure 6a; (**c**) the histograms of B component of Figure 6a; (**d**) the histograms of R component of Figure 6b; (**e**) the histograms of G component of Figure 6b; (**f**) the histograms of B component of Figure 6b.

An image histogram displays the distribution of the values of its pixels and provides some statistical information about the image. The histograms of each component of the color lena image and its cipher image are shown in Figure 7. The experimental results in Figure 7 show objectively the statistical distribution of plaintext and ciphertext pixels. The histogram of the cipher image shows that the pixel distribution in the cipher image is very uniform, which means that our improved algorithm has excellent performance in resisting statistical attacks.

The variance of a histogram can quantitatively describe the distribution of pixel values, which is calculated by [54]

(33) $$\mathrm{var}(Z)=\frac{1}{n^{2}}\sum_{i=1}^{n}\sum_{j=1}^{n}\frac{1}{2}{\left(z_{i}-z_{j}\right)}^{2}.$$

where **Z** is a vector and **Z** = {*z*_1_, *z*_2_, …, *z*_256_}, *z_i_* and *z_j_* are the numbers of pixels with gray values equal to *i* and *j* respectively. The lower value of variance indicates the higher uniformity of ciphered images.

In the experimental tests, the variances of the histograms of the lena plain image (size of 256 × 256 × 3) and its cipher image were calculated by using Equation (33). The results obtained using two different algorithms are listed in Table 1. From Table 1, one can see that the average variance of the cipher image lena obtained with the proposed improved algorithm is 241.4141, which is much less than that of Wang’s algorithm [61]. Thus, our improved algorithm has better performance in resisting statistical attacks.

#### 5.3.2. Correlation of Two Adjacent Pixels

**Figure 8:**
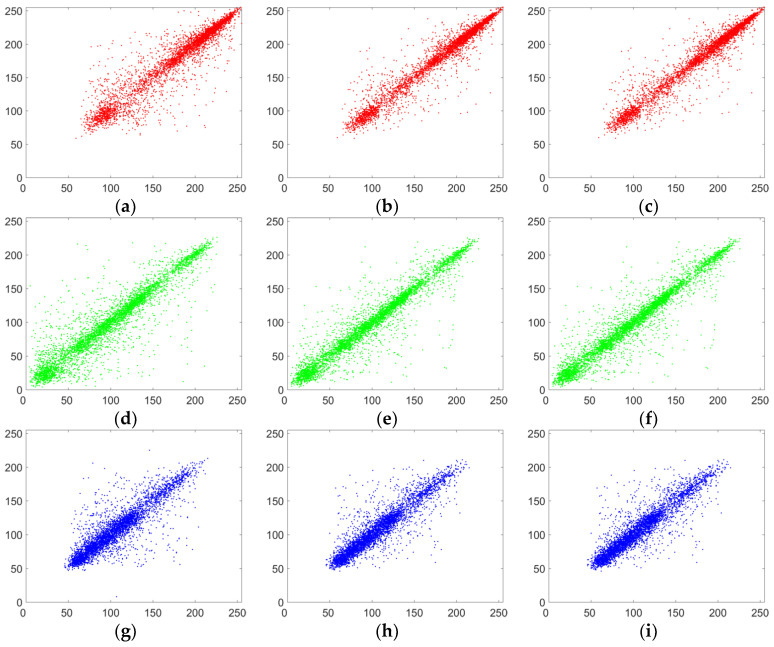
Correlation analysis of the plain image. (**a**) Horizontal correlation in R channel; (**b**) vertical correlation in R channel; (**c**) diagonal correlation in R channel; (**d**) horizontal correlation in G channel; (**e**) vertical correlation in G channel; (**f**) diagonal correlation in G channel; (**g**) horizontal correlation in B channel; (**h**) vertical correlation in B channel; (**i**) diagonal correlation in B channel.

**Figure 9:**
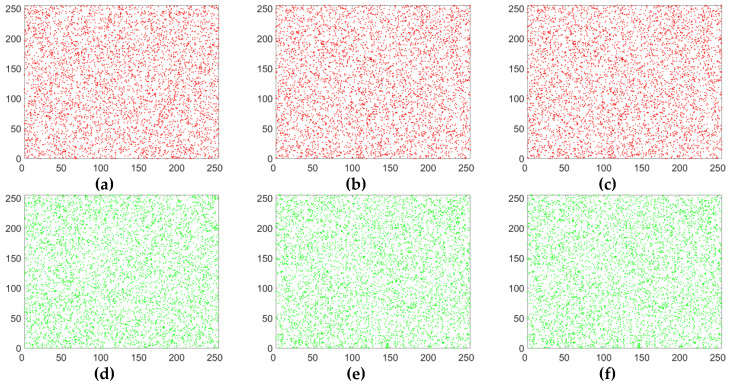

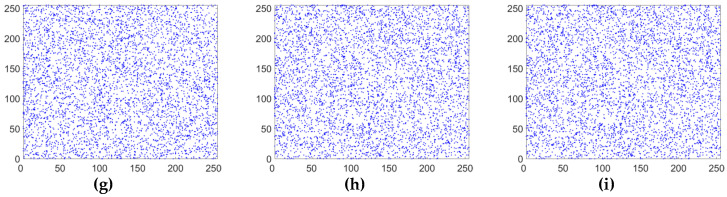
Correlation analysis of the corresponding cipher image. (**a**) Horizontal correlation in R channel; (**b**) vertical correlation in R channel; (**c**) diagonal correlation in R channel; (**d**) horizontal correlation in G channel; (**e**) vertical correlation in G channel; (**f**) diagonal correlation in G channel; (**g**) horizontal correlation in B channel; (**h**) vertical correlation in B channel; (**i**) diagonal correlation in B channel.

Adjacent pixels in images usually have a strong correlation. A good encryption algorithm should break the correlation of adjacent pixels in an image. In order to directly describe the correlation of adjacent pixels in an image, based on 5000 randomly selected pairs of pixels (in horizontal, vertical and diagonal directions), the correlation distribution graphs of the lena plain image and its corresponding cipher image are drawn in Figure 8 and Figure 9. It can be seen that the adjacent pixels in three directions in the plain image have a strong correlation, while those in the cipher image have almost no correlation and it is a random pattern. The results mean that our improved scheme has greatly eliminated the correlation of adjacent pixels. 

In order to quantitatively depict the correlation of adjacent pixels of an image, we introduce correlation coefficient index $r_{XY}$, which is calculated as follows:

(34) $$r_{XY}=\mathrm{cov}\left(X,Y\right)/\sqrt{D\left(X\right)}\sqrt{D\left(Y\right)}\;$$

(35) $$E\left(X\right)=\frac{1}{N}\sum_{i=1}^{N}x_{i}\;$$

(36) $$D\left(X\right)=\frac{1}{N}{\sum_{i=1}^{N}\left(x_{i}-E\left(X\right)\right)}^{2}\;$$

(37) $$\mathrm{cov}\left(X,Y\right)=\frac{1}{N}\sum_{i=1}^{N}\left(x_{i}-E\left(X\right)\right)\left(y_{i}-E\left(Y\right)\right).$$

where X and Y are gray-scale values of two adjacent pixels in the images. For the color lena image, the correlation coefficients of adjacent pixels in R component of plaintext image and R component of ciphertext image were calculated respectively. The results are listed in Table 2. From Table 2, we can see that the correlation coefficients of adjacent pixels in R component of plaintext image are close to 1 while those of the cipher image are close to 0. The experimental results also show that our improved algorithm has smaller absolute values of correlation coefficient than Wang’s algorithm in the vertical and diagonal directions and Pak’s algorithm in all three directions.

#### 5.3.3. Sensitivity Analysis 

In order to resist differential attacks, the algorithm must be sensitive to the secret keys and plain images. To measure the sensitivity of an algorithm to tiny changes in key or plain image, we cite two metrics. One is the number of pixel changing rate (NPCR), another is the unified averaged changed intensity (UACI). The definitions of NPCR and UACI are

(38) $$\mathrm{NPCR}=\frac{1}{m\times n}\sum_{i=1}^{m}\sum_{j=1}^{n}\delta (i,j)\times 100\%,$$

(39) $$\mathrm{UACI}=\frac{1}{m\times n}(\sum_{i=1}^{m}\sum_{j=1}^{n}\frac{\left|c_{1}(i,j)-c_{2}(i,j)\right|}{255})\times 100\%.$$

where *m, n* represent the pixel rows and columns of an image, respectively. Here, **C**_1_ = [ *c*_1_(*i*, *j*)] and **C**_2_ = [*c*_2_(*i*, *j*)] express two encrypted images corresponding to two security keys or two plain images, and *δ*(*i*, *j*) is computed by

(40) $$\delta (i,j)=\{\begin{array}{c} 1,\;if\;c_{1}(i,j)\neq c_{2}(i,j),\\ 0,\;if\;c_{1}(i,j)=c_{2}(i,j). \end{array}\;$$

The desired value of NPCR is 1 and the desired value of UACI is 0.3346 [54].

To measure the sensitivity of our improved algorithm for the plain image, the color lena image (size 256 × 256 × 3) is chosen as the plain image one, and the plain image two is obtained by changing only one pixel of the plain image one. Then, two encrypted images are obtained by executing the improved encryption algorithm with the same secret keys, respectively. NPCR and UACI values are computed with two cipher images, and the results are listed in Table 3. The results indicate that our improved encryption algorithm is very sensitive to the plain image. 

To measure the sensitivity of the improved algorithm to the secret keys, two different keys with a tiny difference are used to encrypt the same plain image lena and the two cipher images, **C**_1_ and **C**_2_, are obtained. The tiny change (10^−14^) is introduced to one of the secret keys (*x*_0_, *u*) while keeping all the others unchanged. Similarly, *k* is changed to *k* + 1, *N*_0_ is changed to *N*_0_ + 1, *C*_0_ is changed to *C*_0_ + 1, while keeping all the others unchanged. The NPCR and UACI of the cipher images **C**_1_ and **C**_2_ are given in Table 4 and Table 5. The experimental results indicate that our improved algorithm is very sensitive to any slight change in each secret key.

#### 5.3.4. Information Entropy Analysis 

Image information entropy is an important way to measure the randomness of the pixel distribution. Let *I* be an image and its information entropy can be calculated as:

(41) $$H(I)=-\sum_{i=0}^{2^{n}-1}P(I_{i})\log_{2}[P(I_{i})],$$

where *P*(*I_i_*) denotes the occurrence probability of gray level *i*, *I_i_ = i*, and *i* = 0, 1, 2, …*,* 2*^n^*. Here, 2*^n^* is the number of grayscale levels of an image. If *P*(*I_i_*) = 1/2*^n^*, then the image is completely random. For an image with 256 gray-scales, *n* = 8 and the image has 2^8^ grayscale levels, so the ideal value of information entropy is 8. For an encrypted image, the closer the entropy is to 8, the closer the image is to a randomly distributed image. We experimentally tested the information entropy of the color lena ciphertext images obtained by three kinds of encryption algorithms. The results of the information entropy corresponding to the R, G and B channels are listed in Table 6. From Table 6, one can see that all the entropy values are significantly closer to 8, so the randomness is satisfactory. Among these three algorithms, our improved algorithm has the largest average entropy value. Hence, our improved encryption scheme is more capable of resisting information entropy-based attacks.

#### 5.3.5. Cropping and Noise Attack

**Figure 10:**
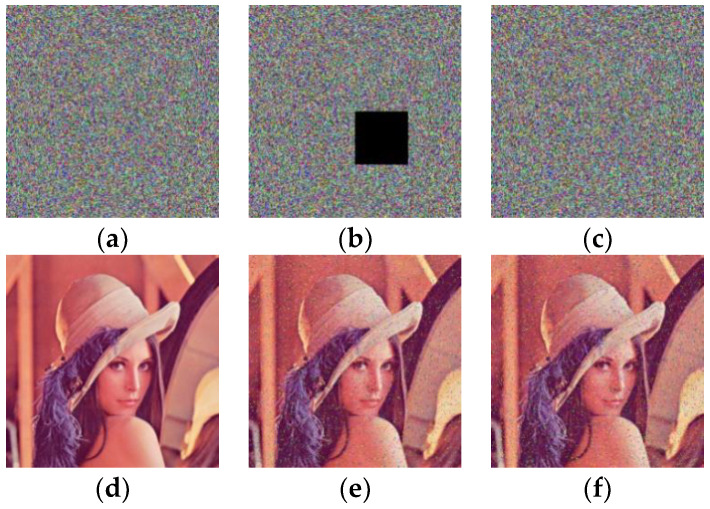
Data loss and noise attack. (**a**) The original cipher image; (**b**) the cipher images with data loss; (**c**) the cipher image added with 3% “salt & pepper” noise; (**d**) the decrypted image of (**a**); (**e**) the decrypted image of (**b**); (**f**) the decrypted image of (**c**).

To test the performance of our improved scheme in resisting data loss and noise attacks. The encrypted lena image (Figure 10a) was attacked by a data cut with a size of 64 × 64 (Figure 10b) and a 3% “salt & pepper” noise attack (Figure 10c), respectively. Then, these cipher images were decrypted respectively and the results of the decryption are given in Figure 10d–f. The results indicate that our improved scheme can resist cutting and noise pollution attacks.

### 5.4. Analysis of Speed

A practical encryption algorithm should be efficient in terms of encryption speed. To test the encryption speed of the improved scheme, three RGB color images with different size have been used for the encryption. The simulation experiments were run on a desktop PC with Intel(R) Core i5-4590 3.30 GHz CPU, 4 GB RAM and 500 GB hard disk. The operating system was 64 bits Microsoft Windows 7 and the computational platform was Matlab R2016b. The average encryption/decryption time taken by Pak’s algorithm, Wang’s algorithm and our improved algorithm for processing the images with different size are shown in Table 7. The results show that our algorithm has the fastest speed. This is because our encryption algorithm has abandoned binary XOR operations.

## 6. Conclusions

In this paper, an improved cryptanalysis on a color image cryptosystem is presented. It has been shown that the equivalent secret key and all the unknown parameters of the cryptosystem can be recovered by our chosen-plaintext attack algorithm. Furthermore, based on the analysis of defects in the original cryptosystem, an improved color image encryption scheme is proposed. The contributions of this paper include two aspects: First, a more complete and efficient method to comprehensively crack Pak’s encryption scheme is proposed, which further enriches the research of cryptanalysis. The validity and correctness of the cryptanalysis algorithm were verified by theoretical analysis and experimental results. Second, a new color image encryption algorithm with a higher security and a higher encryption efficiency is proposed. In the new encryption scheme, the generation of diffusion arrays depends on the content of the image itself and the permutation position array. In the process of diffusion, two effects of ciphertext feedback and pixel scrambling are also implemented simultaneously. Using these methods, the security of the cryptosystem is enhanced. Experimental results and security analysis demonstrate that the improved cryptosystem can achieve a satisfactory security level after two rounds of diffusion encryption. 

Looking to the future in image encryption field, some new research directions are worth considering, such as efficient image encryption technology in the resource-constrained mobile social network [67] or sensor network communication environment [68]. Another interesting form of encryption is searchable encryption [69], which is a very promising direction in the field of cloud computing.

## Figures and Tables

**Table 1:** Variances of the histograms of the Lena image.

Channel	Plain Image	Cipher Image [61]	Cipher Image
R	63,888.1328	527.3242	244.6797
G	28,546.0078	504.7522	239.7656
B	86,487.8906	501.6874	239.7969
Average	57,516.9492	511.2546	241.4141

**Table 2:** Correlation coefficients of the plain image and cipher images of lena in the R channel.

Directions	Plain Image	Cipher Image		
		R	Reference [61]	Ours
H	0.9567	−0.0026	0.00037	0.00063
V	0.9239	−0.0038	−0.00540	−0.00052
D	0.8888	0.0017	0.00166	−0.00012

**Table 3:** Values of NPCR and UACI of Lena cipher images.

Channel	NPCR [61]	NPCR	UACI [61]	UACI
R	0.996413	1	0.334801	0.3341
G	0.996328	1	0.334791	0.3363
B	0.996250	0.9974	0.334558	0.3346

**Table 4:** NPCR of the improved algorithm with a slight change in the secret keys.

Channel	*x*_0_ + 10^−14^	*u* + 10^−14^	*k* + 1	*N*_0_ + 1	*C*_0_ + 1
R	0.9961	0.9960	0.9958	0.9959	0.9961
G	0.9959	0.9964	0.9962	0.9962	0.9961
B	0.9963	0.9961	0.9960	0.9964	0.9961

**Table 5:** UACI of the improved algorithm with a slight change in the secret keys.

Channels	*x*_0_ + 10^−14^	*u* + 10^−14^	*k* + 1	*N*_0_ + 1	*C*_0_ + 1
R	0.3334	0.3356	0.3353	0.3370	0.3344
G	0.3350	0.3355	0.9962	0.3348	0.3348
B	0.3343	0.3355	0.3352	0.3340	0.3337

**Table 6:** Entropies of the encrypted lena image by three encryption schemes.

Channels	Reference [60]	Reference [61]	Ours
R	7.9971	7.9970	7.9973
G	7.9972	7.9965	7.9973
B	7.9974	7.9973	7.9974
Average	7.9972	7.9969	7.9973

**Table 7:** The time cost tests.

Image size	Reference [60]	Reference [61]	Ours
256 × 256	0.5693 s	8.2328 s	0.3873 s
512 × 512	2.2340 s	32.7673 s	1.5145 s
1024 × 1024	8.9055 s	131.6625 s	6.0163 s

## References

[1] Alvarez G., Li S. (2006). Some basic cryptographic requirements for chaos-based cryptosystems. Int. J. Bifurc. Chaos.

[2] Zanette D.H. (1996). Generalized kolmogorov entropy in the dynamics of the multifractal generation. Phys. A Stat. Mech. Appl..

[3] Crutchfield J.P., Packard N.H. (1983). Symbolic dynamics of noisy chaos. Phys. D.

[4] Crutchfield J.P., Feldman D.P. (2003). Regularities unseen, randomness observed: Levels of entropy convergence. Chaos.

[5] Fridrich J. (1998). Symmetric ciphers based on two-dimensional chaotic maps. Int. J. Bifurc. Chaos.

[6] Zhang Y., Xiao D. (2014). An image encryption scheme based on rotation matrix bit-level permutation and block diffusion. Commun. Nonlinear Sci. Numer. Simul..

[7] Zhang Y., Xiao D. (2013). Double optical image encryption using discrete chirikov standard map and chaos-based fractional random transform. Opt. Lasers Eng..

[8] Gan Z.H., Chai X.L., Han D.J., Chen Y.R. (2018). A chaotic image encryption algorithm based on 3-D bit-plane permutation. Neural Comput. Appl..

[9] Hu G., Xiao D., Zhang Y., Xiang T. (2016). An efficient chaotic image cipher with dynamic lookup table driven bit-level permutation strategy. Nonlinear Dyn..

[10] Ye G., Zhao H., Chai H. (2016). Chaotic image encryption algorithm using wave-line permutation and block diffusion. Nonlinear Dyn..

[11] Abd-El-Hafiz S.K., AbdElHaleem S.H., Radwan A.G. (2016). Novel permutation measures for image encryption algorithms. Opt. Lasers Eng..

[12] Li Y., Wang C., Chen H. (2017). A hyper-chaos-based image encryption algorithm using pixel-level permutation and bit-level permutation. Opt. Lasers Eng..

[13] Zhang Y., Xiao D., Shu Y., Li J. (2013). A novel image encryption scheme based on a linear hyperbolic chaotic system of partial differential equations. Signal Process. Image Commun..

[14] Wang X., Liu C., Zhang H. (2016). An effective and fast image encryption algorithm based on chaos and interweaving of ranks. Nonlinear Dyn..

[15] Xu L., Gou X., Li Z., Li J. (2017). A novel chaotic image encryption algorithm using block scrambling and dynamic index based diffusion. Opt. Lasers Eng..

[16] Hua Z., Yi S., Zhou Y. (2018). Medical image encryption using high-speed scrambling and pixel adaptive diffusion. Signal Process..

[17] Huang H., He X., Xiang Y., Wen W., Zhang Y. (2018). A compression-diffusion-permutation strategy for securing image. Signal Process..

[18] Cao C., Sun K., Liu W. (2018). A novel bit-level image encryption algorithm based on 2D-LICM hyperchaotic map. Signal Process..

[19] Chai X. (2017). An image encryption algorithm based on bit level brownian motion and new chaotic systems. Multimed. Tools Appl..

[20] Hua Z., Jin F., Xu B., Huang H. (2018). 2D Logistic-Sine-coupling map for image encryption. Signal Process..

[21] Hua Z., Zhou Y. (2016). Image encryption using 2D Logistic-adjusted-Sine map. Inf. Sci..

[22] Kaur M., Kumar V. (2018). Efficient image encryption method based on improved lorenz chaotic system. Electron. Lett..

[23] Liu J., Yang D., Zhou H., Chen S. (2018). A digital image encryption algorithm based on bit-planes and an improved logistic map. Multimed. Tools Appl..

[24] Zhu C. (2012). A novel image encryption scheme based on improved hyperchaotic sequences. Opt. Commun..

[25] Zhang Y., Tang Y. (2018). A plaintext-related image encryption algorithm based on chaos. Multimed. Tools Appl..

[26] Ye G., Huang X. (2016). A secure image encryption algorithm based on chaotic maps and SHA-3. Secur. Commun. Netw..

[27] Wu X., Kan H., Kurths J. (2015). A new color image encryption scheme based on DNA sequences and multiple improved 1D chaotic maps. Appl. Soft Comput..

[28] Wang X., Zhu X., Wu X., Zhang Y. (2017). Image encryption algorithm based on multiple mixed hash functions and cyclic shift. Opt. Lasers Eng..

[29] Chai X., Gan Z., Zhang M. (2016). A fast chaos-based image encryption scheme with a novel plain image-related swapping block permutation and block diffusion. Multimed. Tools Appl..

[30] Chai X., Chen Y., Broyde L. (2017). A novel chaos-based image encryption algorithm using DNA sequence operations. Opt. Lasers Eng..

[31] Guesmi R., Farah M.A.B., Kachouri A., Samet M. (2016). A novel chaos-based image encryption using DNA sequence operation and secure hash algorithm SHA-2. Nonlinear Dyn..

[32] Hu T., Liu Y., Gong L.-H., Guo S.-F., Yuan H.-M. (2017). Chaotic image cryptosystem using DNA deletion and DNA insertion. Signal Process..

[33] Wang X., Liu C. (2016). A novel and effective image encryption algorithm based on chaos and DNA encoding. Multimed. Tools Appl..

[34] Wang X.-Y., Li P., Zhang Y.-Q., Liu L.-Y., Zhang H., Wang X. (2017). A novel color image encryption scheme using DNA permutation based on the lorenz system. Multimed. Tools Appl..

[35] Wang X.-Y., Zhang Y.-Q., Bao X.-M. (2015). A novel chaotic image encryption scheme using DNA sequence operations. Opt. Lasers Eng..

[36] Zhang L.-M., Sun K.-H., Liu W.-H., He S.-B. (2017). A novel color image encryption scheme using fractional-order hyperchaotic system and DNA sequence operations. Chin. Phys. B.

[37] Zhang D., Liao X., Yang B., Zhang Y. (2018). A fast and efficient approach to color-image encryption based on compressive sensing and fractional fourier transform. Multimed. Tools Appl..

[38] Wang X., Wang Q., Zhang Y. (2015). A fast image algorithm based on rows and columns switch. Nonlinear Dyn..

[39] Tong X.-J., Zhang M., Wang Z., Liu Y., Xu H., Ma J. (2015). A fast encryption algorithm of color image based on four-dimensional chaotic system. J. Vis. Commun. Image Represent..

[40] Liu H., Kadir A., Sun X. (2017). Chaos-based fast colour image encryption scheme with true random number keys from environmental noise. IET Image Process..

[41] Liu W., Sun K., Zhu C. (2016). A fast image encryption algorithm based on chaotic map. Opt. Lasers Eng..

[42] Bibi N., Farwa S., Muhammad N., Jahngir A., Usman M. (2018). A novel encryption scheme for high-contrast image data in the fresnelet domain. PLoS ONE.

[43] Farwa S., Muhammad N., Shah T., Ahmad S. (2017). A novel image encryption based on algebraic s-box and arnold transform. 3D Res..

[44] Farwa S., Shah T., Muhammad N., Bibi N., Jahangir A., Arshad S. (2017). An image encryption technique based on chaotic s-box and arnold transform. Int. J. Adv. Comput. Sci. Appl..

[45] Martin K., Lukac R., Plataniotis K.N. (2005). Efficient encryption of wavelet-based coded color images. Pattern Recognit..

[46] Shahed M.A. (2007). Wavelet based fast technique for images encryption. Basrah J. Sci..

[47] Gao H.J., Zhang Y.S., Liang S.Y., Li D.Q. (2006). A new chaotic algorithm for image encryption. Chaos Solitons Fractals.

[48] Guariglia E. (2016). Entropy and fractal antennas. Entropy.

[49] Guariglia E. (2018). Harmonic sierpinski gasket and applications. Entropy.

[50] Li C., Lin D., Lu J. (2017). Cryptanalyzing an image-scrambling encryption algorithm of pixel bits. IEEE Multimed..

[51] Li C., Liu Y., Xie T., Chen M.Z.Q. (2013). Breaking a novel image encryption scheme based on improved hyperchaotic sequences. Nonlinear Dyn..

[52] Wang X., Luan D., Bao X. (2014). Cryptanalysis of an image encryption algorithm using chebyshev generator. Digit. Signal Prog..

[53] Wu J., Liao X., Yang B. (2018). Cryptanalysis and enhancements of image encryption based on three-dimensional bit matrix permutation. Signal Process..

[54] Zhu C., Sun K. (2018). Cryptanalyzing and improving a novel color image encryption algorithm using rt-enhanced chaotic tent maps. IEEE Access.

[55] Wu X., Zhu B., Hu Y., Ran Y. (2017). A novel colour image encryption scheme using rectangular transform-enhanced chaotic tent maps. IEEE Access.

[56] Zhang W., Yu H., Zhao Y.L., Zhu Z.L. (2016). Image encryption based on three-dimensional bit matrix permutation. Signal Process..

[57] Sun K.H., He S.B., Yin L.Z., Duo L.K. (2012). Application of fuzzyen algorithm to the analysis of complexity of chaotic sequence. Acta Phys. Sin..

[58] Sun K.H., He S.B., He Y., Yin L.Z. (2013). Complexity analysis of chaotic pseudo-random sequences based on spectral entropy algorithm. Acta Phys. Sin..

[59] He S.B., Sun K.H., Zhu C.X. (2013). Complexity analyses of multi-wing chaotic systems. Chin. Phys. B.

[60] Pak C., Huang L. (2017). A new color image encryption using combination of the 1d chaotic map. Signal Process..

[61] Wang H., Xiao D., Chen X., Huang H. (2018). Cryptanalysis and enhancements of image encryption using combination of the 1d chaotic map. Signal Process..

[62] Chen J., Han F., Qian W., Yao Y.-D., Zhu Z.L. (2018). Cryptanalysis and improvement in an image encryption scheme using combination of the 1d chaotic map. Nonlinear Dyn..

[63] Li S., Chen G., Mou X. (2015). On the dynamical degradation of digital piecewise linear chaotic maps. Int. J. Bifurc. Chaos.

[64] Li S., Chen G., Wong K.-W., Mou X., Cai Y. (2004). Baptista-type chaotic cryptosystems: Problems and countermeasures. Phys. Lett. A.

[65] Curiac D.I., Volosencu C. (2012). Chaotic trajectory design for monitoring an arbitrary number of specified locations using points of interest. Math. Probl. Eng..

[66] Curiac D.I., Iercan D., Dragan F., Banias O. Chaos-based cryptography: End of the road?. In Proceedings of the International Conference on Emerging Security Information, System and Technologies.

[67] Zhang S., Wang G., Liu Q., Abawajy J.H. (2018). A trajectory privacy-preserving scheme based on query exchange in mobile social networks. Soft Comput..

[68] Bhuiyan M.Z.A., Wang G., Wu J., Cao J., Liu X., Wang T. (2017). Dependable structural health monitoring using wireless sensor networks. IEEE Trans. Dependable Secur..

[69] Zhang Q., Liu Q., Wang G. (2018). PRMS: A personalized mobile search over encrypted outsourced data. IEEE Access.

